# An analysis of neurovascular disease markers in the hippocampus of *Tupaia chinensis* at different growth stages

**DOI:** 10.3389/fneur.2022.1083182

**Published:** 2023-01-17

**Authors:** Yiqiang Ouyang, Ying Zhang, Xiaoping Guo, Jiafu Li, Qingqing Ao, Songchao Guo, Mingyuan Zhang, Junming Sun

**Affiliations:** ^1^Laboratory Animal Center, Guangxi Medical University, Nanning, China; ^2^Health and Regimen School, Guangxi Vocational and Technical College, School of Food and Biotechnology, Nanning, China

**Keywords:** *Tupaia chinensis*, hippocampal proteomic analysis, different growth stages, neurovascular disease, neurovascular disease biomarkers

## Abstract

**Introduction:**

It is considered that *Tupaia chinensis* can replace laboratory primates in the study of nervous system diseases. To date, however, protein expression in the brain of *Tupaia chinensis* has not been fully understood.

**Method:**

Three age groups of *T. chinensis*-15 days, 3 months and 1.5 years—were selected to study their hippocampal protein expression profiles.

**Results:**

A significant difference was observed between the 15-day group and the other two age groups, where as there were no significant differences between the 3-month and 1.5-year age groups. The Kyoto Encyclopedia of Genes and Genomes (KEGG) analysis found that differentially expressed proteins could be enriched in several pathways related to neurovascular diseases, such as metabolic pathways for Alzheimer's disease (AD), Huntington's disease, Parkinson's disease, and other diseases. The KEGG enrichment also showed that relevant protein involved in oxidative phosphorylation in the hippocampus of *T. chinensis* for 15days were downregulated, and ribosomal proteins (RPs) were upregulated, compared to those in the hippocampus of the other two age groups.

**Discussion:**

It was suggested that when the hippocampus of *T. chinensis* developed from day 15 to 3 months, the expression of oxidatively phosphorylated proteins and RPs would vary over time. Meanwhile, the hippocamppal protein expression profile of *T. chinensis* after 3 months had become stable. Moreover, the study underlines that, during the early development of the hippocampus of *T. chinensis*, energy demand increases while protein synthesis decreases. The mitochondria of *T. chinensis* changes with age, and the oxidative phosphorylation metabolic pathway of mitochondria is closely related to neurovascular diseases, such as stroke and cerebral ischemia.

## Introduction

The hippocampus is a critical component of brain research and memory functions. An injury to the hippocampus is accompanied by many cognitive disorders, and morphological and functional changes in the hippocampus are also associated with pathological changes caused by many diseases of the nervous system (1–3). Animal brains continue to develop after birth, and there are certain differences in the growth characteristics of the hippocampus at different stages of development. These differences involve not only anatomy but also molecular biology, that is, gene protein expression (4–7). An oxidative damage to ribonucleic acid (RNA) increased significantly with age in all regions of the hippocampus (8). It could thus suggest some brain development characteristics and processes, and knowing more about these characteristics would help us understand the formation of brain research and memory functions. As neurovascular diseases are usually related to age, it is necessary to study how their markers change with age.

*Tupaia chinensis* is well suited for scientific study as a closely related phylogenetic primate due to its small size and short breeding cycle. *T. chinensis* has been found to have a larger brain than rodents and a more developed nervous system, making it having a higher application value in the study of neurovascular diseases (9). In recent years, many scholars have used *T. chinensis* to study the markers of neurovascular diseases such as stroke and cerebral ischemia (10–12). *T. chinensis* has a relatively longer life span, exceeding 10 years on average (9, 13). When considering the growth characteristics of *T. chinensis*, most *T. chinensis* used for laboratory experiments are aged between 3 months and 1.5 years, which corresponds to the human age of 10–20 years. Understanding the physiological features of the brain of *T. chinensis* at this growth stage facilitates its use in the study of neurovascular diseases. As tree shrews are more similar to humans than developing rodents and have a significantly higher brain-to-body weight ratio than rats, they are often used in animal models of diseases of the nervous system, such as cerebral ischemia, stroke, and depression.

Proteins are closely related to life and various forms of life activities. We expect to find clues concerning the biological functions of genes, which could be through the study of proteomes and then reveal their role in the whole functional network, thus understanding the phenomena and essence of life. Therefore, proteomics will play an extremely important role in discovering the mystery of life (14, 15). “Proteome” refers to the entire set of proteins expressed by the genome through transcription, translation, and post-translational modifications (16, 17).

Proteomics is a study of the dynamic changes of all proteins expressed by the genome of the cells of an organism in a specific time and space and analyzes the protein composition, the expression patterns, and the relationships among various components of the cell, enabling us to understand the unity of the structure and function of proteins, to further regulate the vital activities of living organisms, and to reveal the nature of life phenomena (18). The present study compared hippocampal protein expression profiles of *T. chinensis* at three different age groups from the perspective of bioinformatics to find proteins that manifest a significant change in the process of neural development and better explain the role of proteins in the development of the nervous system. We also hope to find some age-related markers of neurovascular diseases through this research.

## Materials and methods

### Ethics statement

*Tupaia chinensis*, provided by the Kunming Institute of Zoology of the Chinese Academy of Sciences for this study, are standard laboratory animals artificially bred in captivity. Both the animal transport and the experiment were approved by the Laboratory Animal Ethics Committee of Guangxi Medical University and met the requirements of the national standard GB/T 35892-2018 “Laboratory Animal-Guideline for Ethical Review of Animal Welfare” of the People's Republic of China.

### Animals

In total, nine male *T. chinensis* were purchased from the Kunming Institute of Zoology, Chinese Academy of Sciences and divided into three age groups: 15 days, 3 months, and 1.5 years. All were in good health with no abnormalities, and their microorganisms and parasites met the laboratory animal standards of Yunnan province. All *T. chinensis* were rapidly decapitated to obtain their tissue, and hippocampal tissues were promptly placed in liquid nitrogen for cryopreservation.

### Protein extraction

For protein extraction, hippocampal tissue samples from *T. chinensis* were combined with 1:50 (W:V) Lysis Buffer (8 M urea, 2 mM ethylenediaminetetraacetic acid (EDTA), 10 mM DTT, and 1% protease inhibitor cocktail) and thoroughly homogenized with a tissue grinder. Samples were sonicated for 3 min and centrifuged at 13,000 × *g* at 4°C for 10 min to remove debris, and then, protein in the supernatant was precipitated with cold acetone for 3 h at −2°C. After centrifugation at 4°C at 12,000 × *g* for 10 min, the protein deposit was redissolved with urea buffer [8 M urea and 100 mM triethylammonium bicarbonate (TEAB)]. Protein concentration was determined using a Modified Bradford Protein Assay kit according to the instructions of the manufacturer, purchased from ABclonal Technology Co., Ltd. Cat No of Antibodies: NDUFA9 Polyclonal Antibody A3196-50 μl, ATP5F1 Polyclonal Antibody A7645-50 μl, NDUFS3 Polyclonal Antibody A8013-50 μl, RPS21 Polyclonal Antibody A18585-50 μl, RPS18 Polyclonal Antibody 50 μl A11687-50 μl, NDUFS1 Polyclonal Antibody A2592-50 μl, RPS23 Polyclonal Antibody A17528-50 μl, NDUFA10 Polyclonal Antibody A10123-50 μl, RPS13 Polyclonal Antibody A15720-50 μl, SOD1 Polyclonal Antibody A0274-50 μl, RPS2 Polyclonal Antibody 50 μl A6728-50 μl, and NDUFB9 A17454-50 μl.

### Trypsin digestion

For digestion, 100 μg of protein from each sample was first reduced with 10 m MDTT at 37°C for 60 min and then alkylated with 25 mM iodoacetamide (IAM) at room temperature for 30 min in the dark. The urea concentration of the protein samples was diluted to < 2 M by adding 100 mM TEAB. The protein pool of each sample was digested with Sequencing Grade Modified Trypsin with the ratio of protein:trypsin = 50:1 mass ratio at 37°C overnight and 100:1 for the second digestion for 4 h.

### Peptide isobaric labeling

After trypsin digestion, the peptide was desalted *via* a Strata X SPE column and vacuum-dried. The peptide was reconstituted in 20 μl of 500 mM TEAB and processed according to the protocol of the manufacturer for the 8-plex iTRAQ kit. Briefly, one unit of iTRAQ reagent was added to the peptide solution after it had been thawed and dissolved in 50 μl of isopropanol. Peptide mixtures were incubated for 2 h at room temperature, pooled, and dried by vacuum centrifugation.

### High-performance liquid chromatography fractionation

Dried and labeled peptides were reconstituted with high-performance liquid chromatography (HPLC) solution A [2% acetonitrile (ACN), pH 10] and then fractionated into fractions by high-pH reversed-phase HPLC using Waters Bridge Peptide BEH C18 (130 Å, 3.5 μm, 4.6 mm × 250 mm). Briefly, peptides were first separated with a gradient of 2–98% ACN with pH 10 at a speed of 0.5 ml/min over 88 min into 48 fractions. Then, the peptides were combined into 16 fractions and dried by vacuum centrifugation. Peptide fractions were desalted using ZipTip C18 according to the instructions of the manufacturer. Finally, the samples were dried under vacuum and kept at −20°C until mass spectrometer (MS) analysis could be performed.

### High-resolution liquid chromatography with tandem mass spectrometry analysis

Then, the experiment was performed by NanoLC 1000 liquid chromatography with tandem mass spectrometry (LC-MS/MS) using a Proxeon EASY-nLC 1000 coupled to Thermo Fisher Q Exactive. Trypsin digestion fractions were reconstituted in 0.1% formic acid (FA) and loaded directly onto a reverse phase precolumn (Acclaim PepMap^®^ 100C18, 3 μm, 100 Å, 75 μm × 2 cm) at 5 μl/min in 100% solvent A (0.1 M acetic acid in water). Furthermore, the peptides that eluted from the trap column were loaded onto a reverse phase analytical column (Acclaim PepMap^®^ RSLC C18, 2 μm, 100 Å, 50 μm × 15 cm). The gradient was composed of an increase from 15 to 35% solvent B (0.1% FA in 98% ACN) over 30 min and from 35 to 98% solvent B for 5 min and kept in 98% ACN for 5 min at a constant flow rate of 300 nl/min on an EASY-nLC 1000 system. The eluent was sprayed *via* an NSI source at an electrospray voltage of 2.0 kV and then analyzed by tandem mass spectrometry (MS/MS) in Q Exactive. The MS was operated in data-dependent mode, automatically switching between MS and MS/MS. Full-scan MS spectra (from *m*/*z* 350 to 1,800) were acquired on the Orbitrap with a resolution of 70,000. Ion fragments were detected in the Orbitrap at a resolution of 17,500, and the 20 most intense precursors were selected for subsequent decision tree-based ion trap HCD fragmentation with a collision energy of 27% in the MS survey scan with dynamic exclusion of 40.0 s.

### Data processing

The resulting MS/MS raw data were searched against the *T. chinensis* proteome database (Taxon identifier: 246,437 including 20,824 protein sequences; Proteome: UP000011518) downloaded from the UniProt database (https://sparql.uniprot.org/) using the SEQUEST software integration in Proteome Discoverer (version 1.3, Thermo Scientific). Trypsin was chosen as the enzyme, and two missed cleavages were allowed. Carbamidomethylation (C) was set as a fixed modification and oxidation (M), and N-terminal acetylation was set as a variable modification. Searches were performed using a peptide mass tolerance of 20 ppm and a product ion tolerance of 0.05 Da, resulting in a false discovery rate (FDR) of 5%.

### Protein functional annotation

Proteins were then classified using the gene ontology (GO) annotation based on three categories: biological processes, cellular components, and molecular functions. The GO annotation proteome was derived from the UniProt-GOA database (http://www.ebi.ac.uk/GOA/). The Kyoto Encyclopedia of Genes and Genomes (KEGG, https://www.kegg.jp/) database was used to annotate the protein pathway. First, KEGG online service tools KAAS (https://www.genome.jp/tools/kaas/) were used to annotate the protein description in the KEGG database. The annotation result was then mapped into the KEGG pathway database using the KEGG online service tools KEGG mapper.

### Functional enrichment

A two-tailed Fisher's exact test was employed to test GO (http://geneontology.org/), the KEGG pathway, and the domain enrichment of differentially expressed proteins against all identified proteins. Correction for multiple hypothesis tests was performed using standard FDR control methods, and a corrected *p* < 0.05 was considered significant.

### Expression-based clustering and enrichment-based clustering for protein groups

Expression-based clustering and functional enrichment-based clustering for different protein groups were used to explore potential relationships between different protein groups at protein function (such as the KEGG pathway). We first collated all protein groups obtained after functional enrichment analysis along with their *p*-values and then filtered out those categories that were enriched for at least one of the protein groups with a *p* < 0.05. This filtered *p*-value matrix was transformed by the function *x* = –log10 (*p*-value). Finally, these *x*-values were *z*-transformed for each functional category. These *z*-scores were then clustered using one-way hierarchical clustering (Euclidean distance, average link age clustering). Cluster membership was visualized *via* a heat map using the “Heat Map” function of the R-package.

### Western blot assays

Total proteins were extracted from the hippocampal samples of *T. chinensis* from different age groups. The concentration of the protein samples was determined using the BCA kit. Protein samples (25 μg/well) were separated by 8–12% sodium dodecyl sulfate–polyacrylamide gel electrophoresis (SDS-PAGE) and transferred to polyvinylidene fluoride (PVDF) membranes. These membranes were sealed at room temperature for 1 h in 5% blocking solution, incubated at 4°C overnight with diluted primary antibodies, then washed three times with TBST (10 min/time), incubated with a second antibody at room temperature for 2 h, washed in the same way as before, and developed by electrochemiluminescence (ECL). Except for anti-tubulin, which was purchased from Beyotime Biotechnology, all primary antibodies were purchased from ABclonal. Goat anti-rabbit IgG-HRP was purchased from ZSGB-BIO Co. Ltd.

## Results

### Screening of the hippocampal protein expression of *T. chinensis* at different growth stages

For samples from all three age groups, Identify Protein Num from the database of 27,080 proteins was 6,287, Quantify Protein Num with reliability *q* < 0.05 was 5,952, Protein Num with FDR = 0.01 was 1,051, identifying the ratio of peptide fragments was 23.4%, and the average protein coverage was 13.2%.

**Figure 1:**
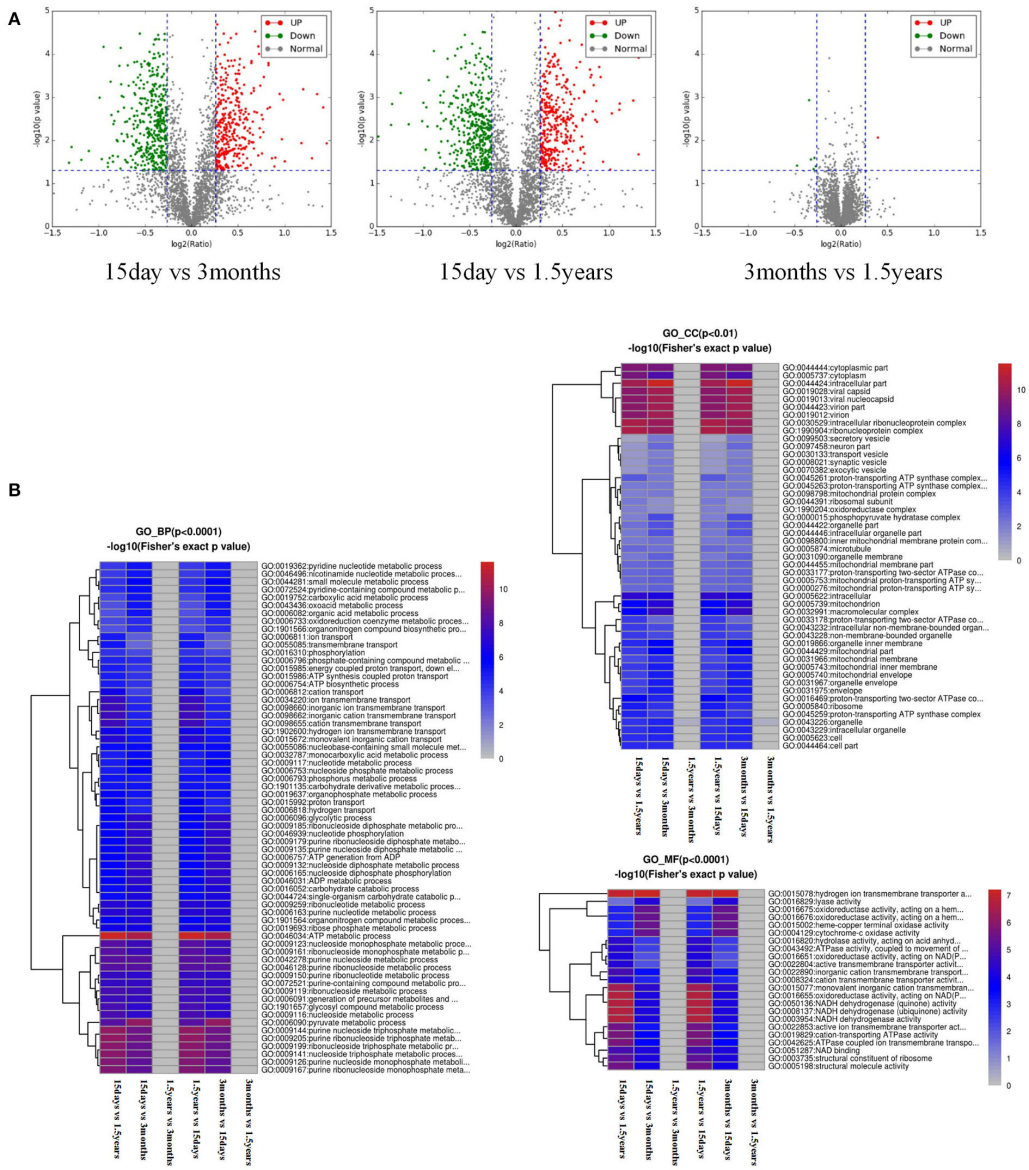
Analysis of a difference in protein expression difference and a functional cluster. **(A)** Differentially expressed proteins and a visible volcano map screened out with the differential multiple of 1.2 and the differential *p*-value of 0.05. The abscisic coordinate in the figure is the multiple variation values of a difference between the two samples, for which log2 logization is performed. The ordinate is the statistical *t*-test *p*-value of the difference in protein expression, which is treated with –log10. The smaller the *p*-value is, the more significant the difference in expression is. Each dot represents a specific protein: green dots represent downregulated proteins, and red dots represent upregulated proteins. There were many different proteins between the 15-day age group and the other two age groups (3-month and 1.5-year age groups), whereas there were only a few different proteins between the 3-month and 1.5-year age groups. **(B)** Functional enrichment-based clustering for protein groups, and functional enrichment of the different proteins in the three biological processes, molecular functions, and cellular components. Reddish color indicates a significant difference; there were obvious differences between the 15-day age group and the other two age groups (3-month and 1.5-year age groups), whereas there was almost no difference between the 3-month and 1.5-year age groups.

The volcano plot showed a relatively significant difference in the hippocampal protein expression between the 15-day age group, the 3-month age group, and the 1.5-year age group and a few differentially expressed proteins between the 3-month and 1.5-year age groups (Figure 1A). Compared with the 1.5-year age group, there were 781 differentially expressed proteins in the 15-day age group, including 365 upregulated and 416 downregulated (Table 1). Compared with the 3-month age group, there were 748 differentially expressed proteins in the 15-day age group, including 345 upregulated and 403 downregulated (when expression multiple > 1.2, *p* < 0.05).

**Table 1:** Quantity sheet of differential proteins between different groups at different multiples and *P* values.

**Multiples**	***P*-value**	**2&0.01**	**2&0.05**	**1.5&0.01**	**1.5&0.05**	**1.3&0.01**	**1.3&0.05**	**1.2&0.01**	**1.2&0.05**
15D/1.5Y	Up	10	13	45	60	152	207	239	365
	Down	6	6	52	76	142	251	258	416
15D/3M	Up	6	11	38	58	137	198	220	345
	Down	0	4	38	68	164	233	258	403
1.5Y/3M	Up	0	0	0	0	0	1	2	7
	Down	0	0	0	0	1	1	1	1

After the functional enrichment-based clustering analysis, in the three categories of level 2: biological processes, cellular components, and molecular functions, there were certain differences in the hippocampal protein function between the 15-day age group, the 3-month age group, and the 1.5-year age group, but there were no differences between the 3-month and 1.5-year age groups (Figure 1B).

**Figure 2:**
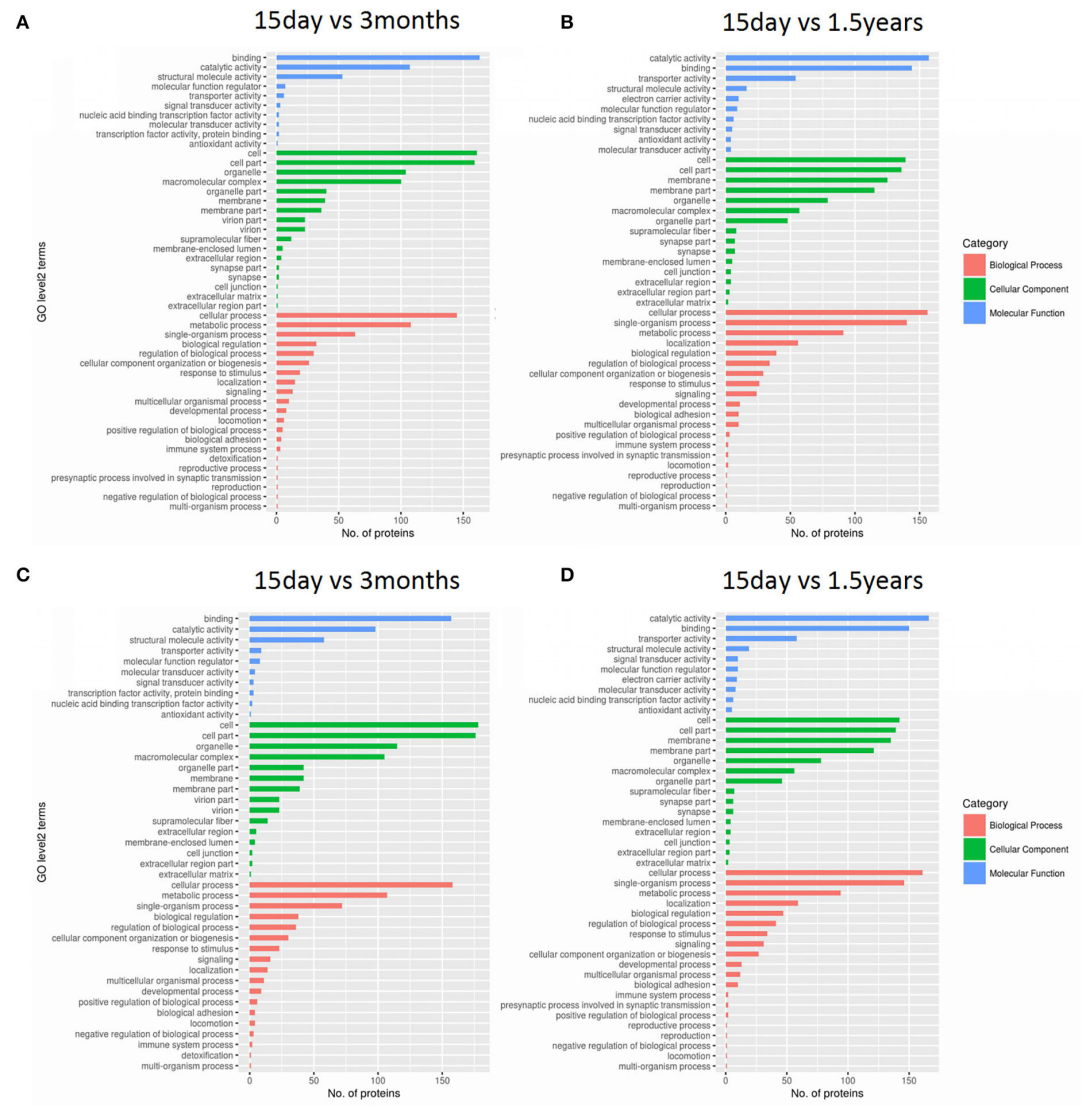
Gene ontology (GO) functional analysis of differentially expressed proteins. Enrichment classification at the second level of GO analysis. The length of the histogram indicates the number of functionally rich proteins, the red stripe represents biological processes, the green stripe represents cellular components, and the blue stripe represents molecular functions. **(A)** The upregulated protein functional enrichment in the 15-day and 1.5-year age groups, **(B)** the upregulated protein functional enrichment in the 15-day and 3-month age groups, **(C)** the downregulated protein functional enrichment in the 15-day and 1.5-year age groups, and **(D)** the downregulated protein functional enrichment in the 15-day and 3-month age groups.

Gene ontology functional significant enrichment analysis of differentially expressed proteins may explain their functional enrichment and illustrate the functional differences of the samples. This analysis used the DAVID software (https://david-d.ncifcrf.gov/) and Fisher's exact test. The GO function was considered to be significantly enriched when the *p* ≤ 0.05. The enrichment analysis figure (Figure 2) of hippocampal protein expression was obtained between the 15-day age group, the 3-month age group, and the 1.5-year age group, and there was no functional enrichment of differentially expressed proteins in the 3-month and 1.5-year age groups. When comparing the 15-day age group with the 3-month age group, there were 65 upregulated and 133 downregulated proteins in biological processes, 29 upregulated and 74 downregulated proteins in cellular components, and 21 upregulated and 98 downregulated proteins in molecular functions. When comparing the 15-day age group with the 1.5-year age group, there were 64 upregulated and 139 downregulated proteins in biological processes, 30 upregulated and 58 downregulated proteins in the cellular component analysis, and 11 upregulated and 100 downregulated proteins in molecular functions.

**Figure 3:**
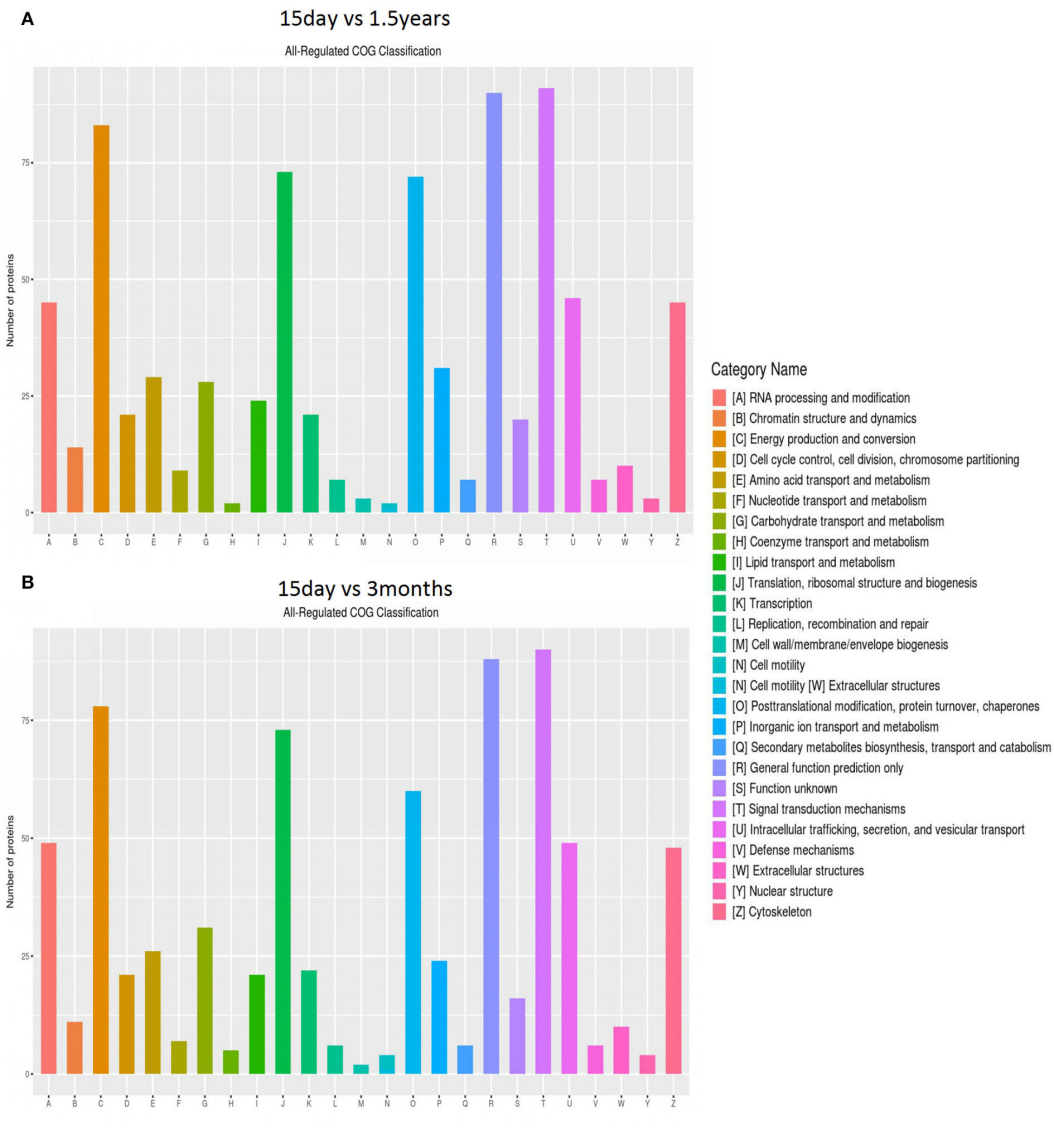
Clusters of orthologous genes (COG) enrichment analysis. The vertical axis represents the number of enriched proteins, and the horizontal axis is an enrichment function entry. **(A)** Functional enrichment of differentially expressed proteins in the 15-day and 1.5-year age groups, and **(B)** functional enrichment of differentially expressed proteins in the 15-day and 3-month age groups.

The distribution and amount of differentially expressed proteins in different clusters of orthologous genes (COG) categories are shown in Figure 3. When comparing the 15-day age group with the 3-month age group, 757 differentially expressed proteins were enriched in 26 COG categories, including 357 upregulated proteins and 400 downregulated proteins. When comparing the 15-day age group with the 1.5-year age group, 783 differentially expressed proteins, including 373 upregulated proteins and 410 downregulated proteins, were enriched in 26 COG categories.

**Figure 4:**
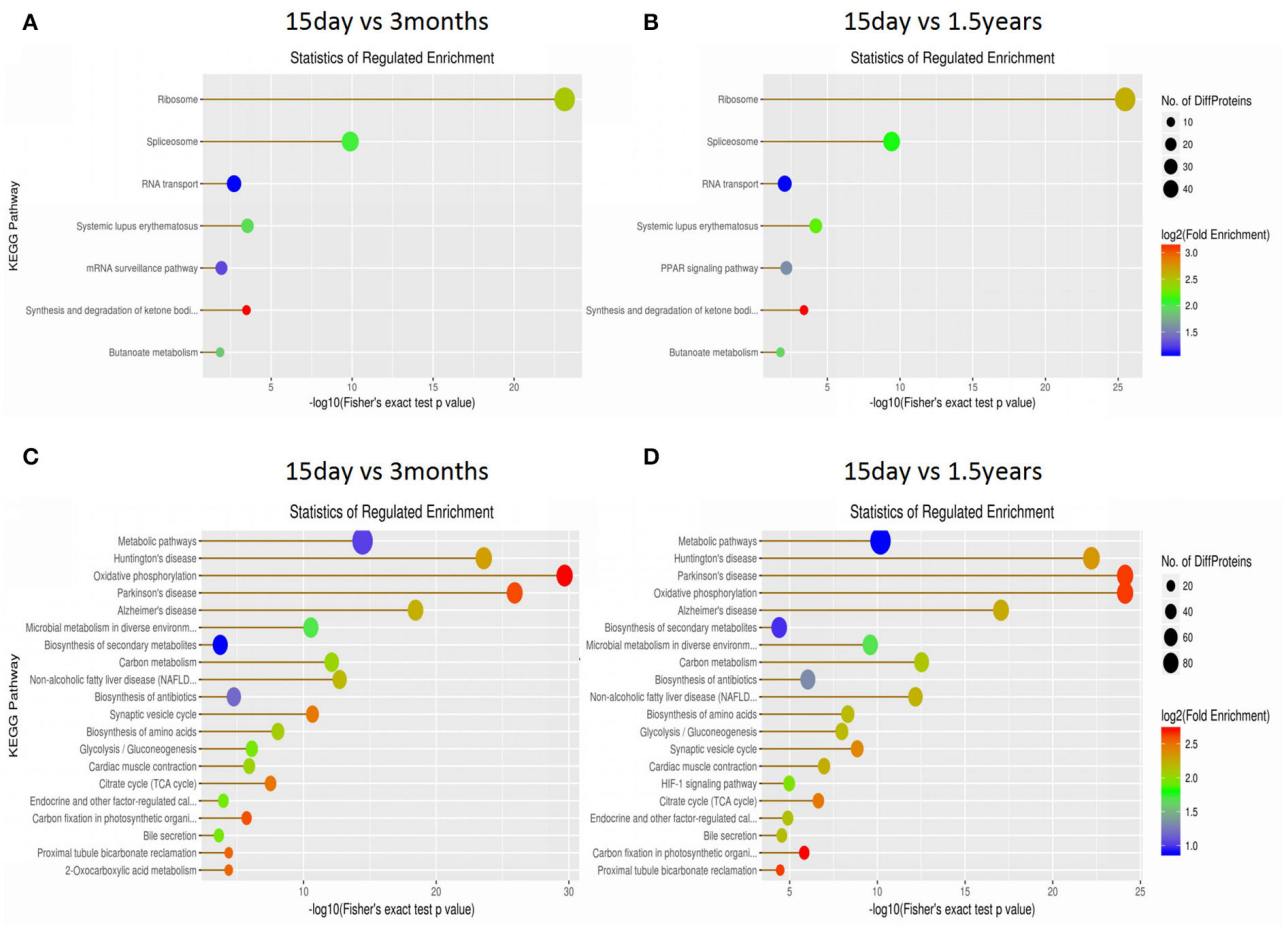
The Kyoto Encyclopedia of Genes and Genomes (KEGG) pathway enrichment analysis of differentially expressed proteins. Each solid circle in the figure is a KEGG pathway term. The horizontal axis represents a significant *p*-value of the KEGG pathway enrichment, which is –log10. The larger the value is, the greater the enrichment is. The ordinate represents the name of the KEGG pathway classification. The size of solid circles represents the number of different proteins under the KEGG pathway classification, and the larger circles represent a larger number of different proteins. The color of the solid circle represents the enrichment multiple of the KEGG pathway classification, and the redder the color is, the greater the enrichment multiple is. **(A)** The upregulated protein pathway enrichment in the 15-day and 3-year age groups. **(B)** The upregulated protein pathway enrichment in the 15-day and 1.5-year age groups. **(C)** The downregulated protein pathway enrichment in the 15-day and 3-year age groups. **(D)** The downregulated protein pathway enrichment in the 15-day and 1.5-year age groups.

The KEGG database was used to classify proteins according to their pathway or function, and an enrichment analysis was performed on the KEGG pathway of differentially expressed proteins in each pair within the three age groups. The enrichment of differentially expressed proteins between the 15-day age group, the 3-month age group, and the 1.5-year age group can be seen in Figure 4 and Supplementary Table S1. When comparing the 15-day age group with the 3-month age group, upregulated proteins were enriched on seven metabolic pathways, whereas downregulated proteins were enriched on 50 metabolic pathways. When comparing the 15-day age group with the 1.5-year age group, upregulated proteins were enriched on seven metabolic pathways, whereas downregulated proteins were enriched on 47 metabolic pathways. Ribosomes and spliceosomes were enriched in significantly upregulated pathways, while the metabolic pathways for Huntington's disease, oxidative phosphorylation, Parkinson's disease, Alzheimer's disease (AD), and other diseases were enriched in the significantly downregulated pathways.

**Figure 5:**
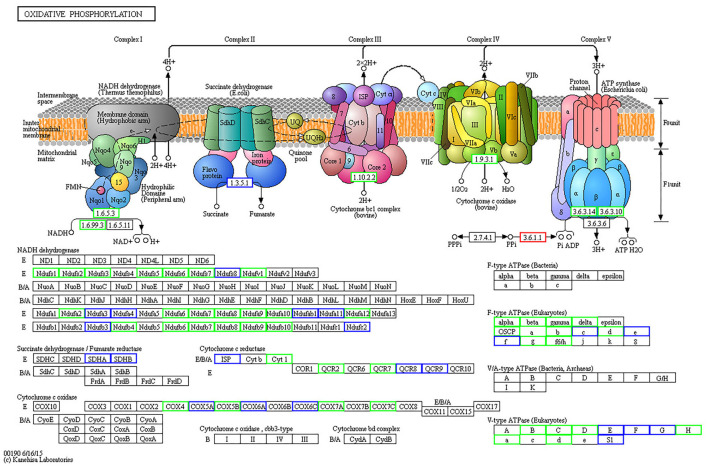
Differentially expressed proteins in oxidative phosphorylation metabolic pathways between the 15-day and 3-month age groups. Differentially expressed proteins are shown in the KEGG pathway map. The rectangular nodes in the figure represent the gene products (such as enzymes or some RNA regulators). Circular nodes represent compounds (that is, substrates or products). Rectangles with rounded corners on a white background represent the other pathways associated with this pathway. Arrows indicate the direction of an enzyme reaction direction or information transfer. The solid line represents a direct action, and the dotted line represents an indirect action. For more details, please refer to: http://www.genome.jp/kegg/document/help_pathway.html. All gene products with a blue border are background proteins, whereas gene products with a white background frame represent the proteins that are not identified in this experiment. The gene products with a red border are upregulated proteins detected in this test, and a green border represents downregulated proteins.

**Figure 6:**
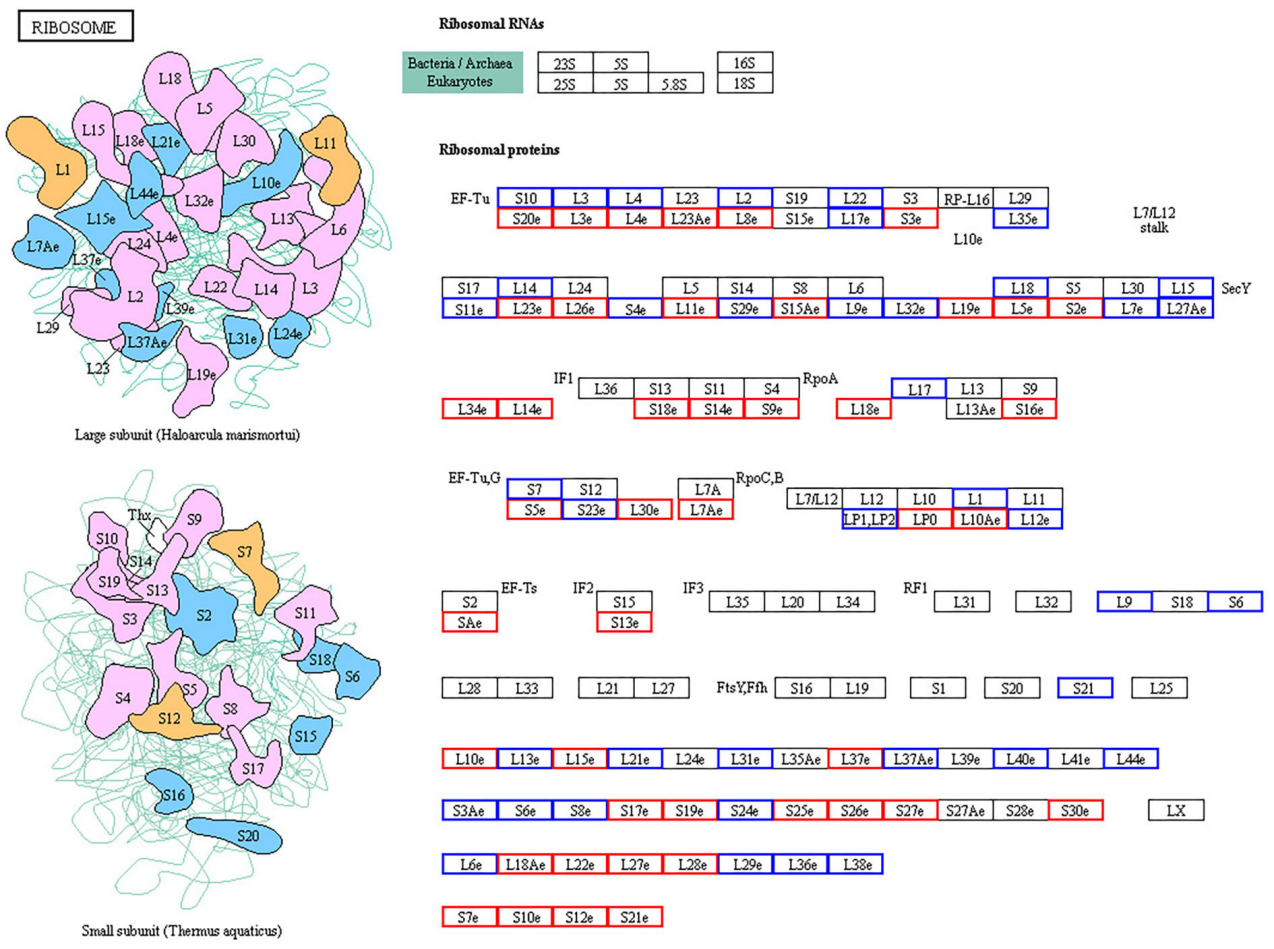
Differentially expressed ribosomal proteins between the 15-day and 3-month age groups. The KEGG pathway chart shows differentially expressed proteins; for more details, please refer to: http://www.genome.jp/kegg/document/help_pathway.html. The gene products in a white background frame represent the proteins that have not been identified in this experiment, the gene products in a blue frame are detected but have no differences, and the gene products in a red frame are upregulated proteins detected in this test.

When comparing the 15-day age group with the 3-month age group, 74 differentially expressed proteins were identified in the oxidative phosphorylation pathway, 40 of which were downregulated (Figure 5, Supplementary Table S2), and 96 proteins were identified in the ribosomal protein (RP) pathway, 43 of which were upregulated (Figure 6, Supplementary Table S3). When comparing the 15-day age group with the 1.5-year age group, 74 differentially expressed proteins were identified in the oxidative phosphorylation pathway, 45 of which were downregulated, and 96 proteins were identified in the RP pathway, 44 of which were upregulated.

### Domain enrichment analysis and subcellular distribution of differentially expressed proteins

**Figure 7:**
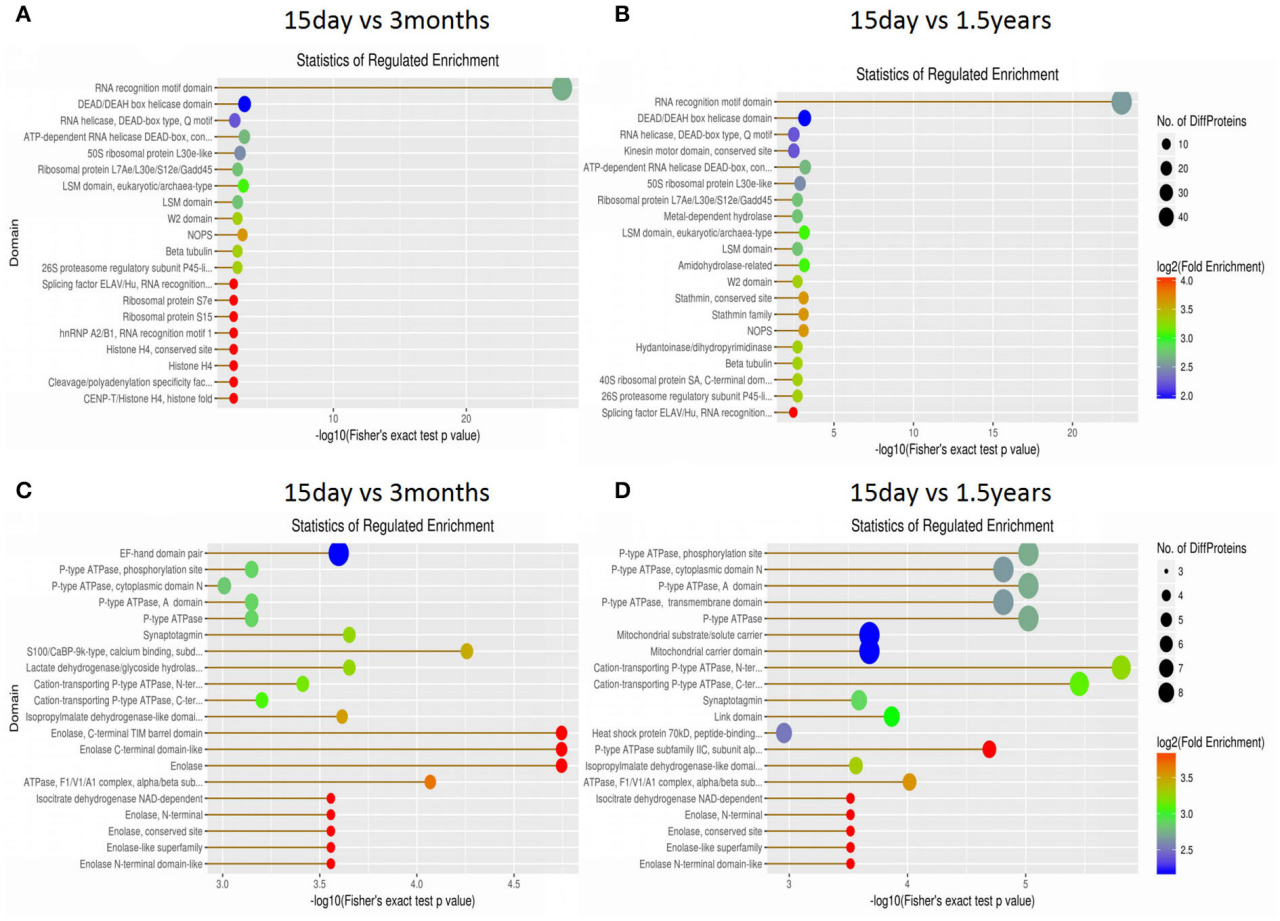
Domain enrichment analysis of differentially expressed proteins. Each solid circle is a domain, and a horizontal coordinate represents a significant *p*-value of domain enrichment, which is –log10. The larger the value is, the more enriched it is. The ordinate represents the name of the domain class. The size of the solid circle represents the number of different proteins under the domain classification, and the larger the circle is, the more different the proteins are. The color of the solid circle indicates the enrichment multiple of the domain classification, and the redder the color, the greater the enrichment multiple. **(A)** The upregulated protein domain enrichment in the 15-day and 3-month age groups. **(B)** The upregulated protein domain enrichment in the 15-day and 1.5-year age groups. **(C)** The downregulated protein domain enrichment in the 15-day and 3-month age groups. **(D)** The downregulated protein domain enrichment in the 15-day and 1.5-year age groups.

The structural domain of the protein was annotated based on sequencing alignment using the InterProScan software tools in the InterPro database (http://www.ebi.ac.uk/interpro/), and domain enrichment analysis of differentially expressed proteins in each pair of age groups was conducted (Figure 7). When comparing the 15-day age group with the 3-month age group, upregulated proteins and downregulated proteins changed in 72 and 126 functional domains, respectively. When comparing the 15-day age group with the 1.5-year age group, upregulated proteins and downregulated proteins changed in 77 and 113 functional domains, respectively.

**Figure 8:**
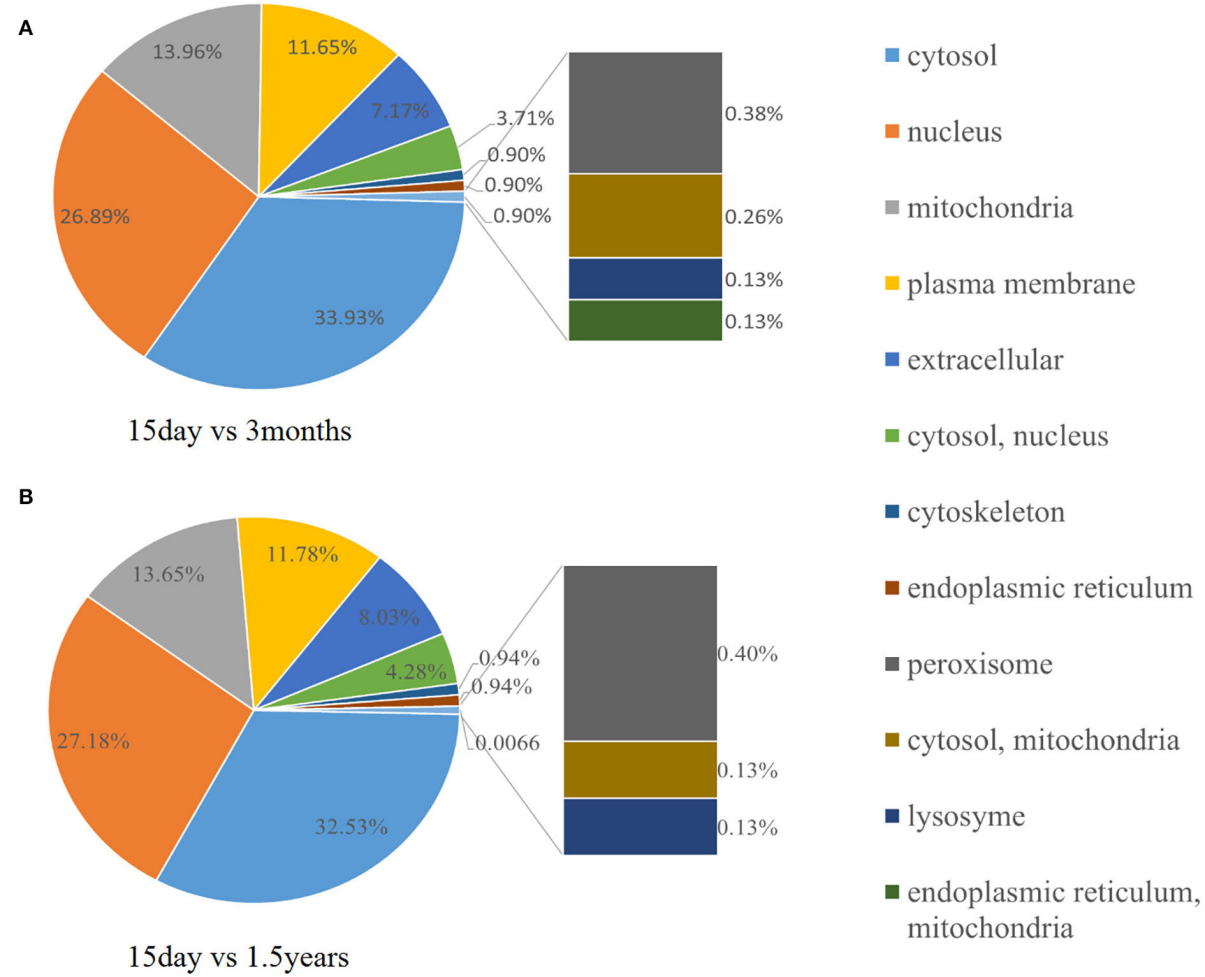
Subcellular distribution of different proteins. The pie chart shows the subcellular distribution of differentially expressed proteins. The fan area represents the percentage of the subcellular distribution in the total amount of differentially expressed proteins. The larger the fan area, the higher the percentage. **(A)** The distribution of different protein subcells in the 15-day and 1.5-year age groups. **(B)** The distribution of different protein subcells in the 15-day and 3-month age groups.

To further observe the distribution of differentially expressed proteins in different subcellular localizations, we counted the number of differentially expressed proteins in each subcellular localization and showed the result in a pie chart (Figure 8). When comparing the 15-day age group with the 3-month age group, differentially expressed proteins were concentrated in 11 subcellular locations, and the six locations with the highest distribution proportion were the cytosol (32.53%), nucleus (27.18%), mitochondria (13.65%), and plasma membrane (11.78%). When comparing the 15-day age group with the 1.5-year age group, differentially expressed proteins were concentrated in 12 subcellular locations, and the six locations with the highest distribution proportion were the cytosol (33.93%), nucleus (26.89%), mitochondria (13.96%), plasma membrane (11.65%), extracellular (7.17%), cytosol, nucleus (3.71%), extracellular (8.03%), and cytosol, nucleus (4.28%).

**Figure 9:**
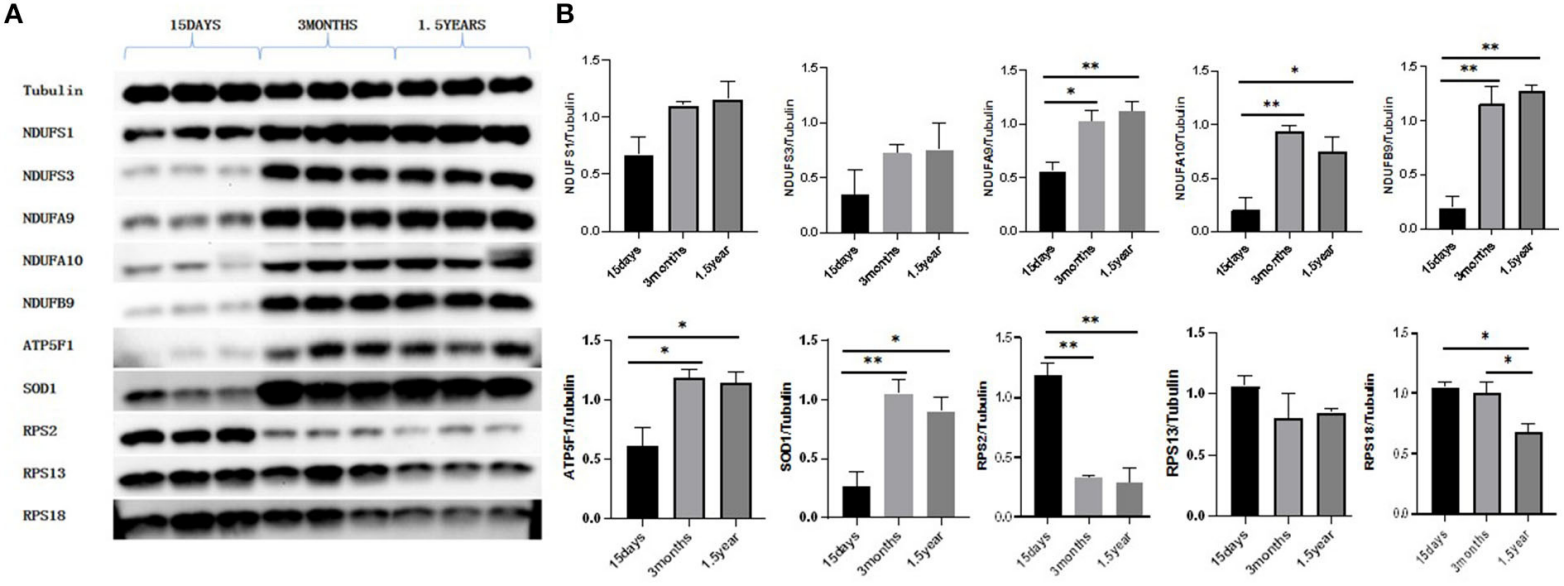
Western blot results. **(A)** Protein expression of NDUFS1, NDUFS3, NDUFA9, NDUFA10, NDUFB9, ATP5F1, SOD1, RPS2, RPS13, and RPS18. **(B)** Gray value analysis results of Western blot. With Tubulin as an internal reference, the expression of proteins related to oxidative phosphorylation pathways, NDUFA9, NDUFA10, NDUFB9, ATP5F1, and SOD1 were significantly lower in the 15-day age group than in the other two age groups. The expression of ribosome-related proteins RPS2 and RPS18 was significantly higher in the 15-day age group than in the other two age groups, which was consistent with the proteomic analysis. *P < 0.05 compared with 15 days group, **P < 0.01 compared with 15 days group.

Western blot showed that the expressions of RPS2 and RPS18 in the ribosome pathway were upregulated at 15 days compared to 3 months and 1.5 years and that NDUFA9, NDUFA10, NDUFB9, ATP5F1, and SOD1 in the oxidative phosphorylation pathway were downregulated at 15 days compared to 3 months and 1.5 years, which was consistent with the sequencing results (Figure 9).

## Discussion

It is very necessary to use animals at appropriate ages for relevant studies in scientific research. *T. chinensis* can live up to 10 years and grow quite rapidly, with sexual maturity at 3–4 months and body maturity at 5–6 months (9). Most *T. chinensis* used for experiment are older than 3 months, and older ones have a higher feeding cost, so older *T. chinensis* of greater months are not usually used unless special requirements are met. The study result indicates that the cerebral hippocampus growth of *T. chinensis* coincides with its stage of sexual maturity, and 3-month-old *T. chinensis* is very similar to mature in hippocampal protein expression, almost the same as 1.5-year-old *T. chinensis*. This will be helpful for the study of the nervous system of *T. chinensis*. The hippocampal protein content in infant *T. chinensis* differs from young and adult *T. chinensis*, which is manifested mainly by fewer oxidatively phosphorylated proteins and higher RPs in infants. There is little difference in the hippocampal protein content of young and adult *T. chinensis*.

Brain development includes not only the increase in the number of cells but also the differentiation and consummation of cells and tissues, as well as functional maturity or neurovascular diseases. It is also accompanied by changes in energy metabolism. Mitochondria, as the energy chamber of cells, generate a lot of energy through oxidative phosphorylation for tissue metabolism, which plays an important role in cell growth, proliferation, differentiation, neurovascular diseases, and other diseases. According to previous research, the functional status of mitochondria determines cell survival to a large extent, and the quantity and quality of mitochondria directly influence the level of energy metabolism, with the former being closely related to the mitochondrial protein (19). Changes in brain mitochondrial protein are not only related to the mitochondrial global function but also related to aging and neurovascular diseases (e.g., Parkinson's disease and AD) (20, 21). Mitochondria, an organ of energy metabolism, provide energy for cell activities through oxidative phosphorylation. Ischemia causes tissue cells to be unable to obtain enough oxygen, meaning that mitochondria cannot effectively carry out oxidative phosphorylation, leading to the pH value falling in the cells and bringing about cellular acidosis and decreased mitochondrial membrane potential. Along with the time extension of ischemia, mitochondrial structure and function would be severely damaged, and cells would eventually die. Several dementia diseases have been related to mitochondrial abnormalities, including AD, Huntington's disease, Parkinson's disease, and other diseases (20–24). Recent research suggested that mitochondria are considered a critical marker of neurovascular diseases such as cerebral ischemia and ischemia/reperfusion injury (25, 26).

Earlier studies determined the oxidase activity of brain, heart, and liver homogenates and cytochrome C in isolated mitochondria and also found that oxidase activity was inhibited in the aging process (27). The present study found that mitochondrial protein expression in the hippocampus of infant *T. chinensis* was lower, and oxidative phosphorylation-related proteins were downregulated at large levels, compared to adults. This, in turn, showed that the improvement of energy metabolism in the process of brain development was a gradual process and that energy metabolism was the lowest in the hippocampus of young animals. It was suggested that damage to the mitochondria of young animals might be conducive to simulating disease models of nervous system abnormalities.

The KEGG pathway analysis showed that differentially expressed proteins were enriched in the pathways of Huntington's disease, oxidative phosphorylation, Parkinson's disease, AD, and other diseases. These genes may be further studied as markers of neurovascular diseases. The main reason for this is that there are many proteins related to oxidative phosphorylation in mitochondria, and the changes in oxidative phosphorylation-related proteins are closely related to the metabolic pathways for Huntington's disease, Parkinson's disease, and AD (28–32).

Ribosomes are the sites of protein synthesis in biological cells. They are composed of RP and ribosomal RNA (rRNA) and are involved in deoxyribonucleic acid (DNA) and protein translation, processing and repair of transcribed and duplicated RNA, and cell proliferation and apoptosis. Ribosomes, as an important organelle in the cell, consist of a 40S small subunit and a 60S large subunit in a ratio of 1:1 in the eucell and have many varieties. Its name is based on the size of the ribosome subunit, where the large subunit RP is named as L1–L44 and the small subunit RP as S1–S31. In AD, the number of ribosomes in neuronal cells is significantly decreased, resulting in impaired protein synthesis, and the large subunit 60S RP L7 can promote apoptosis and regulate cellular transcription by interfering in the expression of cell cycle-related proteins (33, 34).

Mammalian cerebral proteins have a very high level of metabolism. Experiments have proven that more than 90% of rat cerebral proteins have a half-life of only 4–14 days, and some proteins even have a half-life of just several hours (35, 36). Studies showed that newly synthesized cerebral proteins every day during the fetal period account for approximately 47% of total brain proteins, and the rate of synthesis decreases rapidly after birth (37). The expanded repertoire of ribosome biogenesis factors is likely to enable multicellular organisms to coordinate multiple steps of ribosome production in response to different developmental and environmental stimuli (38). The present experiment indicated that the RP level in 15-day-old *T. chinensis* was evidently high compared to that of 3-month-old and 1.5-year-old *T. chinensis*. It has been suggested that *T. chinensis* in this period has a large number of ribosomes in the hippocampus strong protein synthesis and is constantly growing and developing. The level of ribosomes in the hippocampus would gradually decrease with age. Mitochondria gradually increases during hippocampal development, suggesting that hippocampal energy demand increases with age. Ribosomes gradually decrease in the developmental process of the hippocampus, suggesting that some nerve cell proteins are synthesized in large amounts in the early stage of development, and the demand for protein synthesis is reduced after maturity. In the future, more studies on the protein expression profile of different regions of tree shrews, and single-cell sequencing of nerve cells in the brain should be carried out to provide more information for the study of diseases of the nervous system.

## Conclusion

In conclusion, the protein expression level in the hippocampus of 3-month-old *T. chinensis* begins to stabilize. When the hippocampus of *T. chinensis* develops from day 15 to 3 months, the expression levels of oxidatively phosphorylated proteins and RPs vary over time. Meanwhile, the protein expression profile in the hippocampus of *T. chinensis* has become stable within 3 months. In this research, some age-related neurovascular disease markers were found, and a change in their contents was observed in the hippocampus of tree shrews at different ages. Hence, our study provides further insights into animal models of neurovascular diseases in *T. chinensis*.

## Data availability statement

The data presented in the study are deposited in the http://www.proteomexchange.org/ website, accession number PXD039177.

## Ethics statement

The animal study was reviewed and approved by the Experimental Animal Ethics Committee of Guangxi Medical University.

## Author contributions

YZ is responsible for all experimental operations, article writing, data collection, data sorting, statistical analysis, and picture production. MZ is responsible for the experimental operation, data sorting, and statistical analysis. XG is responsible for animal experiment, molecular experiment, and data analysis. QA is responsible for writing articles and sorting data. SG is responsible for guiding experimental ideas, article writing, and article submission. JS is responsible for animal experiments, data collation, and article submission. YO is responsible for guiding experimental ideas, article writing, data collection, data sorting, statistical analysis, picture production, and article submission. All authors contributed to the article and approved the submitted version.
