# Supplementary material for: An analysis of neurovascular disease markers in the hippocampus of Tupaia chinensis at different growth stages
Source: Front Neurol. 2023 Jan 17;13:1083182. doi: 10.3389/fneur.2022.1083182 (PMC9888410; doi:10.3389/fneur.2022.1083182)
Supplement: Supplementary file 1 [file Data_Sheet_1.docx]

Table S1 The difference of protein expression between 15 days group and 1.5 years group (1.5 times content, P < 0.01)

| Description | Regulated-Stage | P0.5-vs-P1.5Y | P0.5-vs-P1.5Y-p |
| --- | --- | --- | --- |
| Pleckstrin homology domain-containing family H member 2 | Up | 4.68672 | 0.002697196 |
| Glutamate receptor delta-1 subunit | Up | 4.61345 | 0.004868246 |
| MARCKS-related protein | Up | 2.96403 | 0.002901392 |
| Fatty acid-binding protein, brain | Up | 2.69613 | 3.94E-05 |
| Voltage-dependent calcium channel subunit alpha-2/delta-4 | Up | 2.49693 | 0.000121338 |
| Neuronal migration protein doublecortin | Up | 2.39792 | 0.001198512 |
| Dihydropyrimidinase-related protein 3 | Up | 2.19029 | 2.27E-05 |
| D-beta-hydroxybutyrate dehydrogenase, mitochondrial | Up | 2.16659 | 0.001411843 |
| Platelet-activating factor acetylhydrolase IB subunit gamma | Up | 2.16107 | 0.001963095 |
| Pleiotrophin | Up | 2.04354 | 0.00237707 |
| Nck-associated protein 5-like protein | Up | 1.88025 | 0.000341466 |
| Serine/threonine-protein kinase DCLK1 | Up | 1.86017 | 0.000199546 |
| Stathmin | Up | 1.82887 | 0.003727415 |
| Pericentrin | Up | 1.82102 | 0.001227446 |
| Basic leucine zipper and W2 domain-containing protein 1 (Fragment) | Up | 1.80602 | 0.003353538 |
| Isocitrate dehydrogenase [NADP] | Up | 1.78419 | 0.000526699 |
| Nuclease-sensitive element-binding protein 1 | Up | 1.77195 | 0.002146109 |
| Fatty acid-binding protein, epidermal | Up | 1.75531 | 0.000304758 |
| 6-phosphogluconolactonase | Up | 1.74405 | 0.000346623 |
| Tenascin | Up | 1.736 | 0.000229299 |
| 3-hydroxy-3-methylglutaryl coenzyme A synthase | Up | 1.71276 | 3.81E-05 |
| Alpha-tubulin N-acetyltransferase 1 | Up | 1.69941 | 0.000106131 |
| 40S ribosomal protein S21 | Up | 1.68147 | 0.002521907 |
| Uncharacterized protein | Up | 1.66452 | 0.000772034 |
| Succinyl-CoA:3-ketoacid-coenzyme A transferase 1, mitochondrial | Up | 1.662 | 0.00072715 |
| Barrier-to-autointegration factor | Up | 1.64329 | 0.003462369 |
| Heterogeneous nuclear ribonucleoprotein A1 (Fragment) | Up | 1.64187 | 0.003203575 |
| Microtubule-associated protein RP/EB family member 1 | Up | 1.63409 | 0.008142255 |
| 40S ribosomal protein S18 | Up | 1.63317 | 6.08E-05 |
| 60S acidic ribosomal protein P1 | Up | 1.61216 | 0.000152245 |
| Dihydropyrimidinase-related protein 1 | Up | 1.59918 | 0.001056976 |
| RNA-binding protein 12 | Up | 1.57089 | 0.004857505 |
| 40S ribosomal protein S13 | Up | 1.56967 | 0.000206937 |
| Serine/arginine-rich splicing factor 7 | Up | 1.56305 | 0.004634241 |
| Thioredoxin domain-containing protein 5 | Up | 1.54607 | 0.005907154 |
| Apolipoprotein A-I | Up | 1.54397 | 0.001100764 |
| Serine/threonine-protein kinase DCLK1 | Up | 1.54173 | 0.000519959 |
| Kinesin-like protein KIF21B | Up | 1.52987 | 0.003990204 |
| High mobility group protein B1 | Up | 1.52941 | 0.002753984 |
| ELAV-like protein | Up | 1.51219 | 0.001451098 |
| Tubulin alpha chain | Up | 1.51012 | 0.002546046 |
| Lipoxygenase homology domain-containing protein 1 | Up | 1.50654 | 0.003049084 |
| RNA-binding protein FUS | Up | 1.50554 | 0.007783026 |
| 40S ribosomal protein S2 | Up | 1.50498 | 0.000858785 |
| Transmembrane protein 39B | Up | 1.50333 | 0.008739119 |
| D-dopachrome decarboxylase | Down | 0.66555 | 0.001901148 |
| Endophilin-A3 | Down | 0.66486 | 0.002310838 |
| Peroxiredoxin-6 | Down | 0.66457 | 6.92E-05 |
| Solute carrier family 2, facilitated glucose transporter member 1 | Down | 0.66444 | 0.000218146 |
| Alpha-1,4 glucan phosphorylase | Down | 0.65463 | 0.0074707 |
| Septin-4 | Down | 0.65434 | 0.006147576 |
| Protein bassoon | Down | 0.65264 | 0.002894679 |
| Tenascin-R | Down | 0.65245 | 0.000563114 |
| Choline transporter-like protein 1 (Fragment) | Down | 0.64844 | 0.005277432 |
| Ras-related protein Rap-2b | Down | 0.64836 | 0.001741335 |
| Complexin-1 | Down | 0.64809 | 0.001050632 |
| Synaptopodin | Down | 0.6465 | 7.55E-05 |
| Ectonucleotide pyrophosphatase/phosphodiesterase family member 6 | Down | 0.641 | 0.000700052 |
| Cysteine and glycine-rich protein 1 | Down | 0.63923 | 0.000707383 |
| Heat shock 70 kDa protein 1-like protein | Down | 0.63865 | 0.00102242 |
| Carbonic anhydrase 4 | Down | 0.63585 | 0.003363011 |
| Malic enzyme | Down | 0.6352 | 0.000108716 |
| NAD-dependent protein deacetylase | Down | 0.62882 | 0.004797306 |
| ATP synthase subunit d, mitochondrial (Fragment) | Down | 0.62876 | 0.000931103 |
| Vesicular glutamate transporter 1 | Down | 0.6273 | 0.002625385 |
| Complexin-2 | Down | 0.62189 | 0.005539691 |
| Synaptotagmin-2 | Down | 0.62056 | 0.001409238 |
| Actin-related protein 6 | Down | 0.61715 | 0.004188299 |
| Beta-enolase | Down | 0.6166 | 0.003588853 |
| Versican core protein | Down | 0.61472 | 0.003634244 |
| Synapsin-1 (Fragment) | Down | 0.61429 | 0.003972145 |
| Nesprin-2 | Down | 0.61355 | 0.001380476 |
| Hyaluronan and proteoglycan link protein 1 | Down | 0.61284 | 0.001435196 |
| Protein Wnt (Fragment) | Down | 0.60694 | 0.001656062 |
| Superoxide dismutase | Down | 0.60038 | 0.000128934 |
| Zinc transporter 3 | Down | 0.59968 | 0.000339358 |
| Carbonic anhydrase 3 | Down | 0.59245 | 0.0032208 |
| Creatine kinase U-type, mitochondrial | Down | 0.58338 | 0.000735085 |
| Protein kinase C and casein kinase substrate in neurons protein 1 | Down | 0.58302 | 6.92E-06 |
| Glutamate decarboxylase 2 | Down | 0.58301 | 0.003535932 |
| Glial fibrillary acidic protein | Down | 0.58153 | 0.00135122 |
| DBF4-type zinc finger-containing protein 2 | Down | 0.57238 | 0.002337117 |
| ATP synthase subunit delta, mitochondrial | Down | 0.56972 | 0.005421687 |
| Pyridoxal kinase (Fragment) | Down | 0.56895 | 0.000911023 |
| Ig gamma-3 chain C (Fragment) | Down | 0.56864 | 0.001321049 |
| Protein EFR3 like protein A | Down | 0.55596 | 0.004715731 |
| Myelin-associated oligodendrocyte basic protein | Down | 0.54208 | 8.52E-05 |
| Tubulin polymerization-promoting protein | Down | 0.52187 | 0.000404265 |
| Calcineurin-binding protein cabin-1 | Down | 0.51689 | 0.004794905 |
| Protein VPRBP | Down | 0.51233 | 0.001717324 |
| Myelin-oligodendrocyte glycoprotein | Down | 0.5012 | 0.001350328 |
| 2',3'-cyclic-nucleotide 3'-phosphodiesterase (Fragment) | Down | 0.49681 | 0.003594834 |
| Rootletin | Down | 0.44906 | 0.004253481 |
| Protein S100-A1 OS=Tupaia chinensis | Down | 0.423 | 0.000802241 |
| Myelin proteolipid protein (Fragment) | Down | 0.39688 | 0.001441155 |
| Myelin basic protein | Down | 0.39376 | 0.004414361 |
| Tetraspanin | Down | 0.35823 | 0.008191449 |

Table S2 Enrichment analysis of KEGG pathway in 15 day group and 1.5 year group

| **Category_Name** | **Diff**  **Mapping** | **Protein**  **Mapping** | **DiffNum** | **Protein**  **Num** | **Fisher's exact test P value** | **FoldEnrichment** |
| --- | --- | --- | --- | --- | --- | --- |
| [Oxidative phosphorylation](http://www.kegg.jp/kegg-bin/show_pathway?map00190) | 46 | 74 | 396 | 2416 | [0.0000](file:///D:\%25E8%25AE%25BA%25E6%2596%2587%25E5%258F%2591%25E8%25A1%25A8\MH180525P02%25E6%25A0%2591%25E9%25BC%25A9%25E6%25B5%25B7%25E9%25A9%25AC%25E8%259B%258B%25E7%2599%25BD%25E8%25B4%25A8%25E7%25BB%2584\Report\Data\P0.5-vs-P1.5Y\Functional_Enrichment\KEGG_IMG_All_Regulated\map00190.html) | 3.79251979 |
| [Parkinson's disease](http://www.kegg.jp/kegg-bin/show_pathway?map05012) | 45 | 74 | 396 | 2416 | [0.0000](file:///D:\%25E8%25AE%25BA%25E6%2596%2587%25E5%258F%2591%25E8%25A1%25A8\MH180525P02%25E6%25A0%2591%25E9%25BC%25A9%25E6%25B5%25B7%25E9%25A9%25AC%25E8%259B%258B%25E7%2599%25BD%25E8%25B4%25A8%25E7%25BB%2584\Report\Data\P0.5-vs-P1.5Y\Functional_Enrichment\KEGG_IMG_All_Regulated\map05012.html) | 3.71007371 |
| [Huntington's disease](http://www.kegg.jp/kegg-bin/show_pathway?map05016) | 48 | 103 | 396 | 2416 | [0.0000](file:///D:\%25E8%25AE%25BA%25E6%2596%2587%25E5%258F%2591%25E8%25A1%25A8\MH180525P02%25E6%25A0%2591%25E9%25BC%25A9%25E6%25B5%25B7%25E9%25A9%25AC%25E8%259B%258B%25E7%2599%25BD%25E8%25B4%25A8%25E7%25BB%2584\Report\Data\P0.5-vs-P1.5Y\Functional_Enrichment\KEGG_IMG_All_Regulated\map05016.html) | 2.84318917 |
| [Ribosome](http://www.kegg.jp/kegg-bin/show_pathway?map03010) | 44 | 96 | 396 | 2416 | [0.0000](file:///D:\%25E8%25AE%25BA%25E6%2596%2587%25E5%258F%2591%25E8%25A1%25A8\MH180525P02%25E6%25A0%2591%25E9%25BC%25A9%25E6%25B5%25B7%25E9%25A9%25AC%25E8%259B%258B%25E7%2599%25BD%25E8%25B4%25A8%25E7%25BB%2584\Report\Data\P0.5-vs-P1.5Y\Functional_Enrichment\KEGG_IMG_All_Regulated\map03010.html) | 2.7962962 |
| [Alzheimer's disease](http://www.kegg.jp/kegg-bin/show_pathway?map05010) | 41 | 90 | 396 | 2416 | [0.0000](file:///D:\%25E8%25AE%25BA%25E6%2596%2587%25E5%258F%2591%25E8%25A1%25A8\MH180525P02%25E6%25A0%2591%25E9%25BC%25A9%25E6%25B5%25B7%25E9%25A9%25AC%25E8%259B%258B%25E7%2599%25BD%25E8%25B4%25A8%25E7%25BB%2584\Report\Data\P0.5-vs-P1.5Y\Functional_Enrichment\KEGG_IMG_All_Regulated\map05010.html) | 2.77934904 |
| [Carbon metabolism](http://www.kegg.jp/kegg-bin/show_pathway?map01200) | 34 | 80 | 396 | 2416 | [0.0000](file:///D:\%25E8%25AE%25BA%25E6%2596%2587%25E5%258F%2591%25E8%25A1%25A8\MH180525P02%25E6%25A0%2591%25E9%25BC%25A9%25E6%25B5%25B7%25E9%25A9%25AC%25E8%259B%258B%25E7%2599%25BD%25E8%25B4%25A8%25E7%25BB%2584\Report\Data\P0.5-vs-P1.5Y\Functional_Enrichment\KEGG_IMG_All_Regulated\map01200.html) | 2.59292929 |
| [Non-alcoholic fatty liver disease (NAFLD)](http://www.kegg.jp/kegg-bin/show_pathway?map04932) | 29 | 67 | 396 | 2416 | [0.0000](file:///D:\%25E8%25AE%25BA%25E6%2596%2587%25E5%258F%2591%25E8%25A1%25A8\MH180525P02%25E6%25A0%2591%25E9%25BC%25A9%25E6%25B5%25B7%25E9%25A9%25AC%25E8%259B%258B%25E7%2599%25BD%25E8%25B4%25A8%25E7%25BB%2584\Report\Data\P0.5-vs-P1.5Y\Functional_Enrichment\KEGG_IMG_All_Regulated\map04932.html) | 2.64073571 |
| [Synaptic vesicle cycle](http://www.kegg.jp/kegg-bin/show_pathway?map04721) | 19 | 37 | 396 | 2416 | [0.0000](file:///D:\%25E8%25AE%25BA%25E6%2596%2587%25E5%258F%2591%25E8%25A1%25A8\MH180525P02%25E6%25A0%2591%25E9%25BC%25A9%25E6%25B5%25B7%25E9%25A9%25AC%25E8%259B%258B%25E7%2599%25BD%25E8%25B4%25A8%25E7%25BB%2584\Report\Data\P0.5-vs-P1.5Y\Functional_Enrichment\KEGG_IMG_All_Regulated\map04721.html) | 3.13295113 |
| [Microbial metabolism in diverse environments](http://www.kegg.jp/kegg-bin/show_pathway?map01120) | 39 | 119 | 396 | 2416 | [0.0000](file:///D:\%25E8%25AE%25BA%25E6%2596%2587%25E5%258F%2591%25E8%25A1%25A8\MH180525P02%25E6%25A0%2591%25E9%25BC%25A9%25E6%25B5%25B7%25E9%25A9%25AC%25E8%259B%258B%25E7%2599%25BD%25E8%25B4%25A8%25E7%25BB%2584\Report\Data\P0.5-vs-P1.5Y\Functional_Enrichment\KEGG_IMG_All_Regulated\map01120.html) | 1.99949070 |
| [Citrate cycle (TCA cycle)](http://www.kegg.jp/kegg-bin/show_pathway?map00020) | 14 | 25 | 396 | 2416 | [0.0000](file:///D:\%25E8%25AE%25BA%25E6%2596%2587%25E5%258F%2591%25E8%25A1%25A8\MH180525P02%25E6%25A0%2591%25E9%25BC%25A9%25E6%25B5%25B7%25E9%25A9%25AC%25E8%259B%258B%25E7%2599%25BD%25E8%25B4%25A8%25E7%25BB%2584\Report\Data\P0.5-vs-P1.5Y\Functional_Enrichment\KEGG_IMG_All_Regulated\map00020.html) | 3.41656565 |
| [Metabolic pathways](http://www.kegg.jp/kegg-bin/show_pathway?map01100) | 122 | 537 | 396 | 2416 | [0.0000](file:///D:\%25E8%25AE%25BA%25E6%2596%2587%25E5%258F%2591%25E8%25A1%25A8\MH180525P02%25E6%25A0%2591%25E9%25BC%25A9%25E6%25B5%25B7%25E9%25A9%25AC%25E8%259B%258B%25E7%2599%25BD%25E8%25B4%25A8%25E7%25BB%2584\Report\Data\P0.5-vs-P1.5Y\Functional_Enrichment\KEGG_IMG_All_Regulated\map01100.html) | 1.38607678 |
| [Biosynthesis of amino acids](http://www.kegg.jp/kegg-bin/show_pathway?map01230) | 21 | 49 | 396 | 2416 | [0.0000](file:///D:\%25E8%25AE%25BA%25E6%2596%2587%25E5%258F%2591%25E8%25A1%25A8\MH180525P02%25E6%25A0%2591%25E9%25BC%25A9%25E6%25B5%25B7%25E9%25A9%25AC%25E8%259B%258B%25E7%2599%25BD%25E8%25B4%25A8%25E7%25BB%2584\Report\Data\P0.5-vs-P1.5Y\Functional_Enrichment\KEGG_IMG_All_Regulated\map01230.html) | 2.61471861 |
| [Spliceosome](http://www.kegg.jp/kegg-bin/show_pathway?map03040) | 26 | 71 | 396 | 2416 | [0.0000](file:///D:\%25E8%25AE%25BA%25E6%2596%2587%25E5%258F%2591%25E8%25A1%25A8\MH180525P02%25E6%25A0%2591%25E9%25BC%25A9%25E6%25B5%25B7%25E9%25A9%25AC%25E8%259B%258B%25E7%2599%25BD%25E8%25B4%25A8%25E7%25BB%2584\Report\Data\P0.5-vs-P1.5Y\Functional_Enrichment\KEGG_IMG_All_Regulated\map03040.html) | 2.23417271 |
| [2-Oxocarboxylic acid metabolism](http://www.kegg.jp/kegg-bin/show_pathway?map01210) | 8 | 13 | 396 | 2416 | [0.0003](file:///D:\%25E8%25AE%25BA%25E6%2596%2587%25E5%258F%2591%25E8%25A1%25A8\MH180525P02%25E6%25A0%2591%25E9%25BC%25A9%25E6%25B5%25B7%25E9%25A9%25AC%25E8%259B%258B%25E7%2599%25BD%25E8%25B4%25A8%25E7%25BB%2584\Report\Data\P0.5-vs-P1.5Y\Functional_Enrichment\KEGG_IMG_All_Regulated\map01210.html) | 3.75446775 |
| [Carbon fixation in photosynthetic organisms](http://www.kegg.jp/kegg-bin/show_pathway?map00710) | 9 | 16 | 396 | 2416 | [0.0003](file:///D:\%25E8%25AE%25BA%25E6%2596%2587%25E5%258F%2591%25E8%25A1%25A8\MH180525P02%25E6%25A0%2591%25E9%25BC%25A9%25E6%25B5%25B7%25E9%25A9%25AC%25E8%259B%258B%25E7%2599%25BD%25E8%25B4%25A8%25E7%25BB%2584\Report\Data\P0.5-vs-P1.5Y\Functional_Enrichment\KEGG_IMG_All_Regulated\map00710.html) | 3.43181818 |
| [Cardiac muscle contraction](http://www.kegg.jp/kegg-bin/show_pathway?map04260) | 15 | 37 | 396 | 2416 | [0.0004](file:///D:\%25E8%25AE%25BA%25E6%2596%2587%25E5%258F%2591%25E8%25A1%25A8\MH180525P02%25E6%25A0%2591%25E9%25BC%25A9%25E6%25B5%25B7%25E9%25A9%25AC%25E8%259B%258B%25E7%2599%25BD%25E8%25B4%25A8%25E7%25BB%2584\Report\Data\P0.5-vs-P1.5Y\Functional_Enrichment\KEGG_IMG_All_Regulated\map04260.html) | 2.47338247 |
| [Endocrine and other factor-regulated calcium reabsorption](http://www.kegg.jp/kegg-bin/show_pathway?map04961) | 12 | 29 | 396 | 2416 | [0.0011](file:///D:\%25E8%25AE%25BA%25E6%2596%2587%25E5%258F%2591%25E8%25A1%25A8\MH180525P02%25E6%25A0%2591%25E9%25BC%25A9%25E6%25B5%25B7%25E9%25A9%25AC%25E8%259B%258B%25E7%2599%25BD%25E8%25B4%25A8%25E7%25BB%2584\Report\Data\P0.5-vs-P1.5Y\Functional_Enrichment\KEGG_IMG_All_Regulated\map04961.html) | 2.52455590 |
| [Glycolysis / Gluconeogenesis](http://www.kegg.jp/kegg-bin/show_pathway?map00010) | 16 | 46 | 396 | 2416 | [0.0017](file:///D:\%25E8%25AE%25BA%25E6%2596%2587%25E5%258F%2591%25E8%25A1%25A8\MH180525P02%25E6%25A0%2591%25E9%25BC%25A9%25E6%25B5%25B7%25E9%25A9%25AC%25E8%259B%258B%25E7%2599%25BD%25E8%25B4%25A8%25E7%25BB%2584\Report\Data\P0.5-vs-P1.5Y\Functional_Enrichment\KEGG_IMG_All_Regulated\map00010.html) | 2.12209046 |
| [Proximal tubule bicarbonate reclamation](http://www.kegg.jp/kegg-bin/show_pathway?map04964) | 7 | 13 | 396 | 2416 | [0.0021](file:///D:\%25E8%25AE%25BA%25E6%2596%2587%25E5%258F%2591%25E8%25A1%25A8\MH180525P02%25E6%25A0%2591%25E9%25BC%25A9%25E6%25B5%25B7%25E9%25A9%25AC%25E8%259B%258B%25E7%2599%25BD%25E8%25B4%25A8%25E7%25BB%2584\Report\Data\P0.5-vs-P1.5Y\Functional_Enrichment\KEGG_IMG_All_Regulated\map04964.html) | 3.28515928 |
| [Biosynthesis of antibiotics](http://www.kegg.jp/kegg-bin/show_pathway?map01130) | 36 | 139 | 396 | 2416 | [0.0022](file:///D:\%25E8%25AE%25BA%25E6%2596%2587%25E5%258F%2591%25E8%25A1%25A8\MH180525P02%25E6%25A0%2591%25E9%25BC%25A9%25E6%25B5%25B7%25E9%25A9%25AC%25E8%259B%258B%25E7%2599%25BD%25E8%25B4%25A8%25E7%25BB%2584\Report\Data\P0.5-vs-P1.5Y\Functional_Enrichment\KEGG_IMG_All_Regulated\map01130.html) | 1.580117724 |

**Note**: KEGG terms is KEGG functional classification, DiffMapping is number of differentially expressed protein in a KEGG term, DiffNum is all the number of differentially expressed protein in all KEGG terms, ProteinMapping is number of quantitative protein in a KEGG term, ProteinNum is all the number of quantitative protein in all KEGG terms.

Table S3 The difference of protein expression on oxidative phosphorylation pathway between 15 day group and 1.5 year group (multiple > 1.2, p-value < 0.01)

| Protein Description | Regulated-Stage | multiples | P-value |
| --- | --- | --- | --- |
| NADH dehydrogenase [ubiquinone] 1 beta subcomplex subunit 9 | Down | 0.78438 | 0.00731171 |
| V-type proton ATPase subunit d 1 | Down | 0.69224 | 0.002282837 |
| NADH dehydrogenase [ubiquinone] 1 alpha subcomplex subunit 7 | Down | 0.79882 | 0.002701413 |
| NADH dehydrogenase [ubiquinone] 1 beta subcomplex subunit 4 | Down | 0.77626 | 0.002467425 |
| NADH-ubiquinone oxidoreductase 75 kDa subunit, mitochondrial | Down | 0.81212 | 0.003995707 |
| NADH dehydrogenase [ubiquinone] 1 beta subcomplex subunit 10 | Down | 0.78039 | 0.003871052 |
| NADH dehydrogenase [ubiquinone] 1 alpha subcomplex subunit 8 | Down | 0.68993 | 0.000884206 |
| V-type proton ATPase subunit B, brain isoform | Down | 0.78099 | 0.0028534 |
| NADH dehydrogenase [ubiquinone] 1 beta subcomplex subunit 7 | Down | 0.75227 | 0.005873199 |
| NADH dehydrogenase [ubiquinone] 1 alpha subcomplex subunit 9, mitochondrial | Down | 0.74577 | 0.004349285 |
| NADH dehydrogenase [ubiquinone] 1 alpha subcomplex subunit 10, mitochondrial | Down | 0.77319 | 0.000988586 |
| Cytochrome c oxidase subunit 7A-related protein, mitochondrial | Down | 0.77153 | 0.001090658 |
| NADH dehydrogenase [ubiquinone] iron-sulfur protein 3, mitochondrial | Down | 0.80944 | 0.00506904 |
| NADH dehydrogenase [ubiquinone] 1 alpha subcomplex subunit 2 | Down | 0.7485 | 0.007900608 |
| Cytochrome b-c1 complex subunit 2, mitochondrial | Down | 0.74519 | 0.002466793 |
| Cytochrome c oxidase subunit 4 isoform 1, mitochondrial | Down | 0.8196 | 0.008819748 |
| ATP synthase subunit beta | Down | 0.80117 | 0.001884373 |
| NADH dehydrogenase [ubiquinone] 1 alpha subcomplex subunit 6 | Down | 0.71132 | 0.001885498 |
| ATP synthase subunit g, mitochondrial | Down | 0.73347 | 0.008185061 |
| Cytochrome c1, heme protein, mitochondrial (Fragment) | Down | 0.77009 | 0.005393185 |
| Cytochrome c oxidase subunit 5B, mitochondrial | Down | 0.82655 | 0.000130865 |
| ATP synthase subunit alpha | Down | 0.71961 | 0.002203139 |
| NADH dehydrogenase [ubiquinone] iron-sulfur protein 5 | Down | 0.77933 | 0.001647075 |
| ATP synthase-coupling factor 6, mitochondrial | Down | 0.82334 | 0.002676746 |
| ATP synthase subunit b, mitochondrial | Down | 0.81045 | 0.003362854 |
| ATP synthase subunit d, mitochondrial (Fragment) | Down | 0.62876 | 0.000931103 |
| NADH dehydrogenase [ubiquinone] 1 beta subcomplex subunit 8, mitochondrial | Down | 0.73394 | 0.00161516 |
| V-type proton ATPase subunit C | Down | 0.82552 | 0.002157964 |
| Inorganic pyrophosphatase | Up | 1.40914 | 0.000593162 |
| NADH dehydrogenase [ubiquinone] 1 beta subcomplex subunit 6 | Down | 0.75351 | 0.005378282 |
| ATP synthase subunit delta, mitochondrial | Down | 0.56972 | 0.005421687 |
| ATP synthase subunit d, mitochondrial | Down | 0.71569 | 0.001556269 |
| V-type proton ATPase subunit H | Down | 0.76689 | 0.002529618 |
